# Blood Oxidative Stress Modulates Alveolar Bone Loss in Chronically Stressed Rats

**DOI:** 10.3390/ijms21103728

**Published:** 2020-05-25

**Authors:** Micaele Maria Lopes Castro, Priscila Cunha Nascimento, Deiweson Souza-Monteiro, Sávio Monteiro Santos, Mayra Barros Arouck, Vinicius Barreto Garcia, Raimundo Fernandes de Araújo, Aurigena Antunes de Araujo, Gabriela de Souza Balbinot, Fabrício Mezzomo Collares, Cassiano Kuchenbecker Rosing, Marta Chagas Monteiro, Cristiane Socorro Ferraz Maia, Rafael Rodrigues Lima

**Affiliations:** 1 Laboratory of Functional and Structural Biology, Institute of Biological Sciences, Federal University of Para, Belem 66075-900, PA, Brazil; micaelecastro@hotmail.com (M.M.L.C.); priscilacunha.n28@gmail.com (P.C.N.); deiwesonmonteiro@gmail.com (D.S.-M.); 2 Laboratory of In Vitro Assays, Immunology and Microbiology, Faculty of Pharmacy, Institute of Biological Sciences, Federal University of Pará, Belem 66075-900, PA, Brazil; saviomontsan@gmail.com (S.M.S.); auriprinino@gmail.com (A.A.d.A.); martachagas2@yahoo.com.br (M.C.M.); 3 Laboratory of Pharmacology of Inflammation and Behavior, Faculty of Pharmacy, Institute of Health Science, Federal University of Pará, Belem 66075-900, PA, Brazil; mayraarouckbarros@gmail.com (M.B.A.); crismaia@ufpa.br (C.S.F.M.); 4 Cancer and Inflammation Research Laboratory, Department of Morphology, Federal University of Rio Grande do Norte, Natal 59078-970, RN, Brazil; araujojr@cb.ufrn.br (V.B.G.); araujojr.morfologia@gmail.com (R.F.d.A.J.); 5 Post-Graduation Programme in Health Science, Federal University of Rio Grande do Norte, Natal 59072-970, RN, Brazil; 6 Post-Graduation Programme in Structural and Functional Biology, Federal University of Rio Grande do Norte, Natal 59072-970, RN, Brazil; 7 Department Biophysics and Pharmacology, Post-Graduation Programme in Public Health, Post-Graduation Programme in Pharmaceutical Science, Federal University of Rio Grande do Norte, Natal 59072-970, RN, Brazil; 8 Dental Materials Laboratory, Faculty of Dentistry, Federal University of Rio Grande do Sul, Porto Alegre 90040-060, Brazil; gabi_balbinot@hotmail.com (G.d.S.B.); fabricio.collares@ufrgs.br (F.M.C.); 9 Department of Periodontology, Faculty of Dentistry, Federal University of Rio Grande do Sul, Porto Alegre 90040-060, Brazil; ckrosing@hotmail.com

**Keywords:** chronic stress, experimental periodontitis, alveolar bone loss, ligatures

## Abstract

We aimed to investigate the effects of chronic stress (CS) on experimental periodontitis (EP) in rats. For this, 28 Wistar rats were divided into four groups: control, ligature-induced experimental periodontitis (EP), chronic stress (CS; by physical restraint model) and CS+EP (association of chronic stress and ligature-induced periodontitis). The experimental period lasted 30 days, including exposure to CS every day and ligature was performed on the 15th experimental day. After 30 days, the animals were submitted to the behavioral test of the elevated plus maze (EPM). Next, rats were euthanized for blood and mandible collection in order to evaluate the oxidative biochemistry (by nitric oxide (NO), reduced-glutathione activity (GSH), and thiobarbituric acid reactive substance levels (TBARS)) and alveolar bone characterization (by morphometric, micro-CT, and immunohistochemistry), respectively. The behavioral parameters evaluated in EPM indicated higher anxiogenic activity in the CS and CS+EP, groups, which is a behavioral reflex of CS. The results showed that CS was able to change the blood oxidative biochemistry in CS and CS+EP groups, decrease GSH activity in the blood, and increase the NO and TBARS concentrations. Thus, CS induces oxidative blood imbalance, which can potentialize or generate morphological, structural, and metabolic damages to the alveolar bone.

## 1. Introduction

The term “stress” is characterized by the unfavorable effect of environmental factors on the physiological functions of the body [1]. Pathologically, when this stressful stimulus is prolonged on the hypothalamus–pituitary–adrenal axis (HPA), it is called chronic stress (CS) [2,3]. An elevated secretion of cortisol that affects the organism homeostasis then occurs, leading to various pathological conditions including hyperglycemia and alteration of cytokine and growth factors levels. These changes may damage various body systems and increase the risk for infections, cardiovascular disease, and immunological disorders [4,5].

In this context, studies indicate that elevated cortisol levels modulate immunological and inflammatory processes that potentiate systemic damages to the organism [6,7]. Besides for these response pathways to CS, the scientific literature suggests that this condition can promote systemic oxidative imbalance by increasing reactive oxygen species (ROS) or generating deficiency in the antioxidant system [8].

In the oral cavity, studies have demonstrated the impact of chronic stress on periodontal tissues [9,10]. Epidemiological surveys and clinical trials identify chronic stress as a potentiator of the aggravation of periodontal diseases, especially periodontitis [11]. Although the literature shows an association between these conditions and the vascular system as a link of an increased inflammatory mediators in the blood [12,13], the mechanisms involved, especially concerning systemic oxidative imbalance, have not been elucidated yet.

In this way, our hypothesis to be tested is that CS is a grievance factor of a blood oxidative stress trigger, which is capable of promoting alveolar bone loss in the presence or absence of induced experimental periodontitis.

## 2. Results

### 2.1. Chronic Immobilization Stress Promotes Anxiogenic-Like Effects in Rats Exposed or Not to Experimental Periodontitis

**Figure 1:**
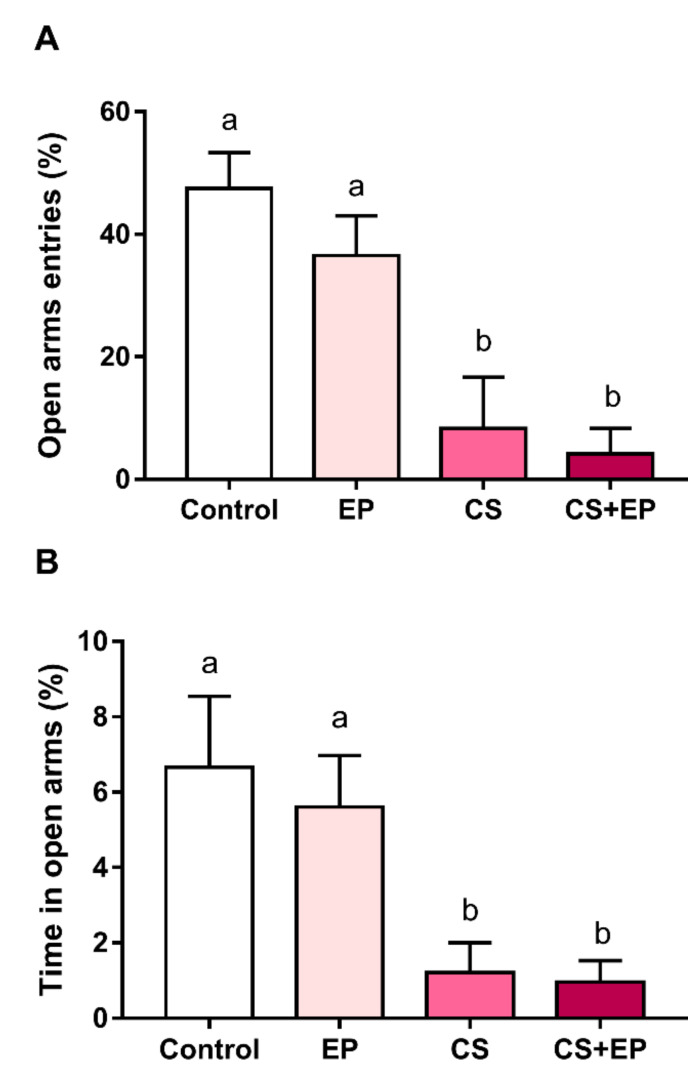
Effects of experimentally induced chronic immobilization stress and ligature-induced periodontitis on anxiety-like behavior of male Wistar rats (90-days-old, *n* = 28). (**A**) Entrance frequency in open arms (%) and (**B**) time in the open arms (%) in the elevate plus maze; results are expressed as mean ± standard error. One-way ANOVA and Tukey’s post-hoc test, *p* < 0.05. Similar overwritten letters did not show statistically significant differences. EP: ligature-induced experimental periodontitis group; CS: chronic stress by physical restraint model; CS+EP: association of chronic stress and ligature-induced periodontitis.

Anxiety was assessed by the elevated plus-maze assay (Figure 1A,B). Groups exposed to stress demonstrated a statistically significantly different pattern compared to non-stressed animals in both open arms entries (F (3, 19) = 11.49, *p* < 0.01, Figure 1A, Table S1) and time in open arms (F (3, 24) = 7.81, *p* < 0.01, Figure 1B, Table S1).

### 2.2. Effects of Chronic Stress and Periodontitis Induced Experimentally on Blood Biochemical Parameters of Adult Rats

**Figure 2:**
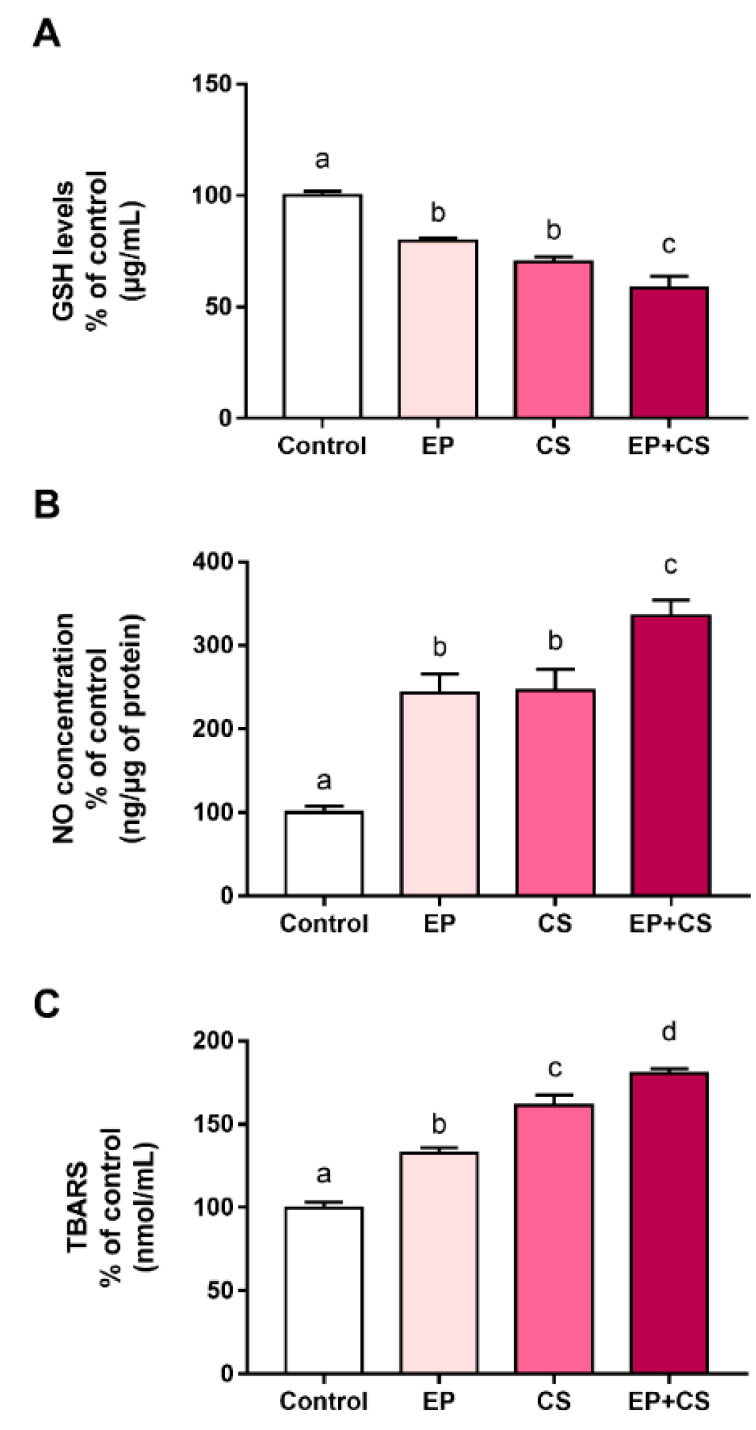
Effects of experimentally induced chronic immobilization stress and ligature-induced periodontitis on oxidative biochemical paraments of male Wistar rats (90-days-old, *n* = 28) in the blood. Results are expressed as mean ± standard error of mean of control percentage of (**A**) glutathione (GSH) levels; (**B**) nitric oxide (NO) levels; and (**C**) lipid peroxidation (TBARS). One-way ANOVA and Tukey’s post-hoc test, *p* < 0.05. Similar overwritten letters do not reveal statistically significant differences. EP: ligature-induced experimental periodontitis group; CS: chronic stress by physical restraint model; CS+EP: association of chronic stress and ligature-induced periodontitis.

As observed in Figure 2, the exposure to induced-chronic stress and experimental periodontitis also generated an imbalance of blood oxidative biochemical parameters. Both exposure to chronic stress and to ligature-induced periodontitis decreased oxidative parameters related to glutathione (GSH) levels when compared to the control group. A synergic effect was observed in animals exposed to both models (F (3, 24) = 36.21, *p* < 0.0001, Figure 2A, Supplementary Materials Table S1).

Furthermore, an increase of nitric oxide (NO) in the blood of animals exposed to chronic stress or periodontitis in comparison to the control group was observed, highlighting the potential effect of the NO levels rising due to the association between experimental chronic stress and periodontitis (F (3, 37) = 31.96, *p* < 0.0001, Figure 2B, Supplementary Materials Table S1).

The effects of experimentally-induced chronic stress and experimental periodontitis on lipid peroxidation were observed by increasing levels of TBARS (thiobarbituric acid reactive substance levels); this effect was potentialized by the association between models, as compared to the control group (F (3, 36) = 91.6, *p* < 0.0001, Figure 2C, Supplementary Materials Table S1).

### 2.3. Induced Chronic Stress is Associated with Changes on Alveolar Bone in Rats Exposed or Not to Experimental Periodontitis

**Figure 3:**
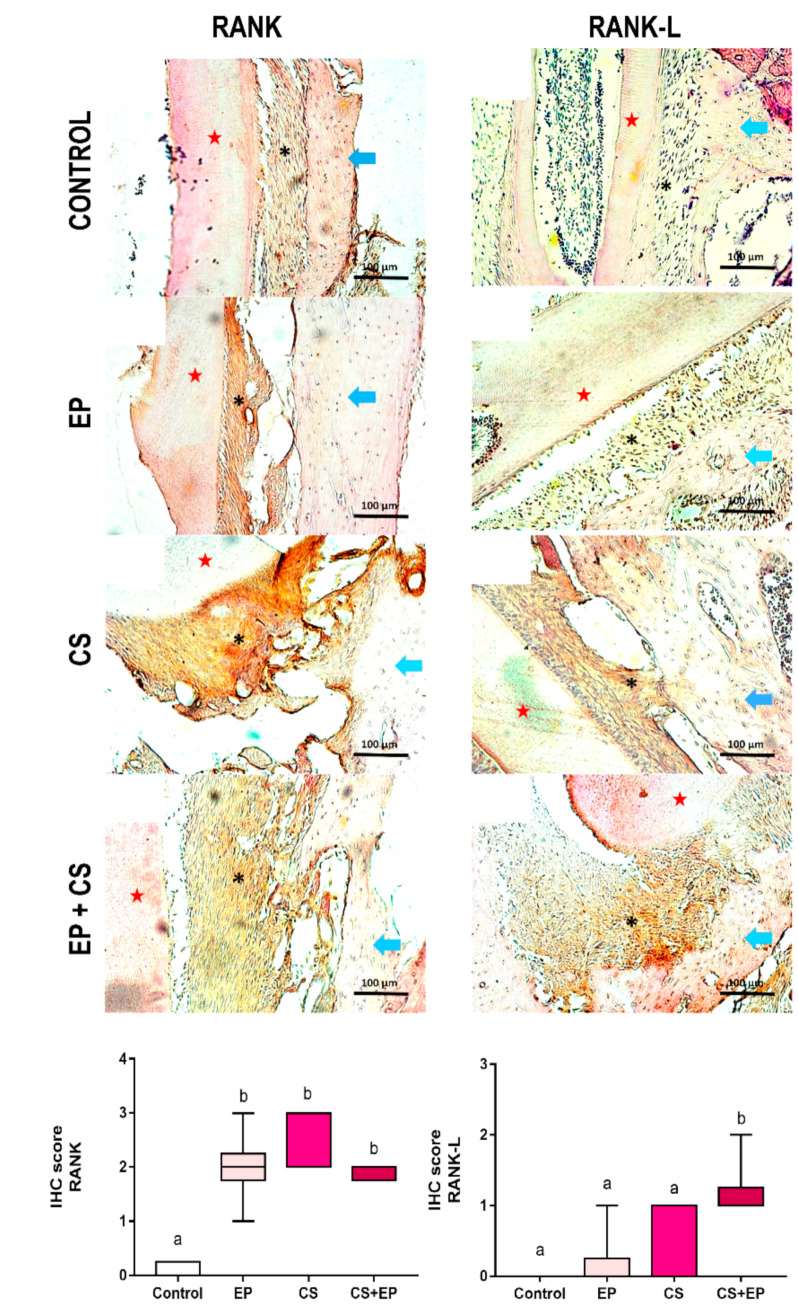
Effects of experimentally induced chronic immobilization stress and ligature-induced periodontitis on RANK and RANK-L expression of the alveolar bone (blue arrows) of male Wistar rats (90-days-old, *n* = 28). Representative photomicrographs of the control group; experimental periodontitis group (EP); chronic stress group (CS); and chronic stress and experimental periodontitis group (CS+EP). Results are expressed as mean ± standard error of immunohistochemistry score (IHC score) of RANK and RANKL expression. Kruskal–Wallis test, *p* < 0.05. Similar overwritten letters did not reveal statistically significant differences. EP: ligature-induced experimental periodontitis group; CS: chronic stress by physical restraint model; CS+EP: association of chronic stress and ligature-induced periodontitis. Scale bar: 100 µm. Cementum (★) and periodontal ligament (*).

In the immunohistochemical analysis, periodontal tissue from groups exposed to chronic stress or experimental periodontitis models demonstrated strong immunostaining for NF-κB (RANK) and receptor activator of the NF-κB ligand (RANKL) (*p* < 0.001, Figure 3, Table S1).

**Figure 4:**
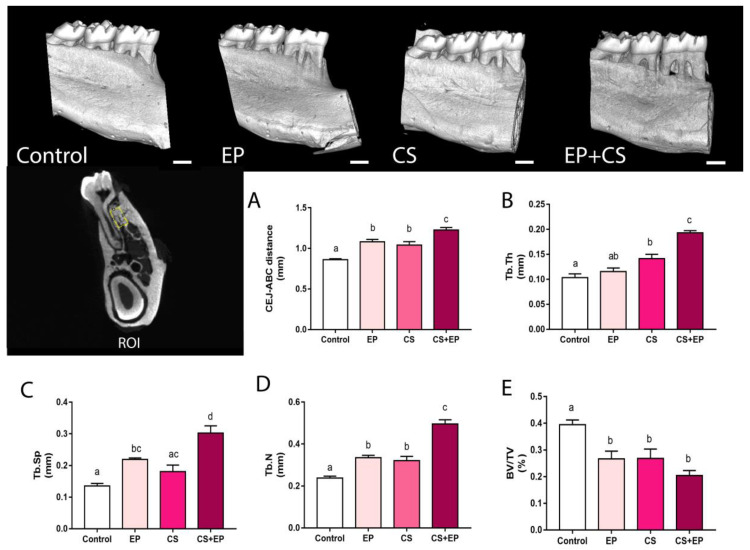
Effects of experimentally induced chronic immobilization stress and ligature-induced periodontitis on the quality of the alveolar bone of male Wistar rats (90-days-old, *n* = 28). Representative three-dimensional images of hemimandibles of the control group; experimental periodontitis group (EP); chronic stress group (CS); and chronic stress and periodontitis group (CS+EP). The representative image of the interradicular region, close to the furcation area, chosen to the standardized region of interest (ROI) for the analyses. Results are expressed as mean ± standard error for micro-CT parameters: (**A**) distance between the cemento-enamel junction (CEJ) and the alveolar bone crest (ABC) in millimeters (mm); (**B**) trabecular thickness (Tb.Th; mm); (**C**) trabecular spacing (Tb.Sp; mm); (**D**) mean number of trabecular per unit length (Tb.N); and (**E**) the percentage of bone volume in relation to the total measured area (BV/TV; %). One-way ANOVA and Tukey’s post-hoc test, *p* < 0.05. Similar overwritten letters did not reveal statistically significant differences. Scale bar: 1 mm.

**Figure 5:**
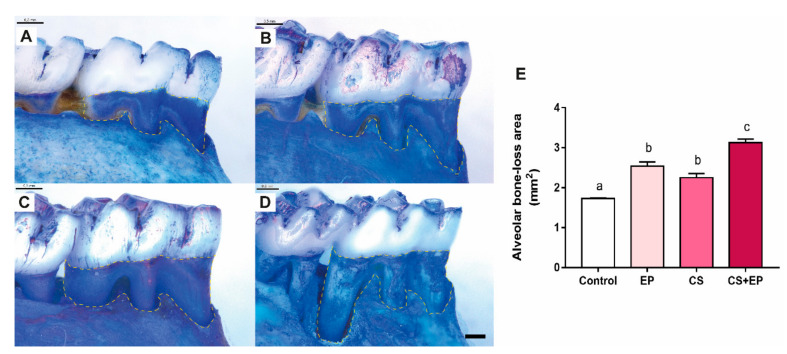
Effects of experimentally induced chronic immobilization stress and ligature-induced periodontitis on the alveolar bone of male Wistar rats (90-days-old, *n* = 28). Representative photomicrographs of hemimandibles of the (**A**) control group; (**B**) experimental periodontitis group (EP); (**C**) chronic stress group (CS), and (**D**) chronic stress and experimental periodontitis group (CS+EP). Results are expressed as mean ± standard error of the mean of (**E**) alveolar bone-loss area (highlighted in yellow). One-way ANOVA and Tukey’s post-hoc test, *p* < 0.05. Similar overwritten letters do not show statistically significant differences. EP: ligature-induced experimental periodontitis group; CS: chronic stress by physical restraint model; CS+EP: association of chronic stress and ligature-induced periodontitis. Scale bar: 0.5 mm.

In the qualitative analyses of alveolar bone by micro-CT, exposure to chronic stress associated or not to experimental periodontitis resulted in the increased of trabecular thickness (Tb.Th; F (3, 19) = 34.4, *p* < 0.0001, Figure 5A, Supplementary Materials Table S1); trabecular spacing (Tb.Sp; F (3, 19) = 17.01, *p* < 0.0001, Figure 4B, Supplementary Materials Table S1); and mean of the number of trabecular per unit length, in relation to the control-animals group (Tb.N; F (3, 19) = 43.65, *p* < 0.0001, Figure 4C, Supplementary Materials Table S1). In addition, groups exposed to chronic stress and/or experimental periodontitis models presented reduction of the percentage of bone volume in relation to the total measured area when compared to the control group (BV/TV; F (3, 14) = 11.54, *p* < 0.0001, Figure 4D, Table S1).

Results of the morphological measures indicated that induced chronic stress led to damage on the alveolar bone of rats exposed or not to experimental periodontitis. In the quantitative analyses by stereomicroscope, a higher alveolar bone-loss area was observed in groups exposed both to chronic stress or experimental periodontitis when compared to control animals (F (3, 40) = 31.8, *p* < 0.0001, Figure 5A–C, Supplementary Materials Table S1). Moreover, chronic stress exacerbated bone loss in comparison to the other groups (Figure 5D, Table S1), as well as when the distance in millimeters between the cementoenamel junction and the alveolar bone crest was used as indicative of alveolar bone loss, as shown in Figure 4A (F (3, 36) = 39.88, *p* < 0.0001, Table S1).

## 3. Discussion

This study showed that the systemic damage caused by chronic stress can modulate the dynamics of alveolar bone resorption, both in the absence of experimental periodontitis and in its presence. In general, the data indicate that CS promotes behavioral changes and generates systemic oxidative damage, such as increasing the amount of nitric oxide and decreasing the activity of the GSH concentration, as well as modifying the RANK and RANK-L system and generating and increasing alveolar bone loss in rats.

Stress is defined as a normal organic response on the body, which, if excessive (CS), strains the capacity of adaptive processes beyond their limits [14]. This condition affects many aspects of physiology, while emotional status and the ways of coping with stress can also influence health and disease [15]. In this way, stress is an important factor in the etiology and maintenance of many inflammatory diseases, including periodontal diseases [16].

Studies that evaluate the effect of chronic stress on periodontal breakdown, in general, demonstrated that stress modulates alveolar bone loss by increasing it [17,18]. On the other hand, this modulation, depending on the stress model, especially intensity, might decrease that condition [19].

The damage caused by chronic stress at the systemic level is intense, acting on several organs and systems. Our study applied a model of chronic, physical, and restrictive stress in rats for 30 days, which triggered behavioral changes, promoting behavior of the anxiogenic type, which is a behavioral reflex of chronic stress [20], demonstrating that the chronic behavioral stress protocol was successful in the exposed groups (i.e., CS and CS+EP groups).

In a periodontal context, the study of the effects of CS becomes important to understand the beginning and progression of periodontitis, whereas psychosomatic condition is able to modulate systemic endocrine and inflammatory pathways that in turn are directly related to susceptibility of periodontal tissues [10,21]. Additionally, the understanding of different conditions that modulate inflammation are important to generate hypotheses in other diseases/conditions. Studying periodontal disease in animals is one of the reliable ways to quantify the effects of the inflammatory process, more than only quantifying the process itself.

Based on the literature, CS is able to modulate the periodontium, through both the HPA axis (increase in cortisol levels) and the autonomic nervous system (increase in noradrenaline and adrenaline secretion) [22]. The systemic oxidative pathway has never been elucidated in this association, either in preclinical or clinical studies. Therefore, this study is the first to present this pathway as a possibility of enhancing damage to the alveolar bone in a condition of chronic stress.

Regarding biological changes in our study, we highlight a systemic oxidative imbalance in animals, especially due to the higher concentration of nitric oxide and decreased concentration of the antioxidant enzyme GSH. This balance is important for the organism, and is involved in mechanisms of inflammatory reactions or acts as a second messenger in several cellular functions [23]. Therefore, when an oxidative imbalance occurs, either in the formation or removal of free radicals, the physiological maintenance of the cell can be affected, enabling cell death [24]. In periodontal disease, such imbalance has the potential of increasing loss of supporting tissues. Thus, ROS cause tissue damage by a variety of different mechanisms, such as DNA and protein damage, oxidation of important enzymes, stimulation of proinflammatory cytokine release and lipid peroxidation oxygen, reactive oxygen species, and tissue damage [25].

Nitric oxide radicals are abundant in several biological processes, acting on the immune system and promoting vasodilatations in the endothelium [26]. This mediator is widely linked to increased osteoclastic activity and also to pro-inflammation, favoring damage to periodontal tissues. However, the reactions promoted by these harmful radicals, such as nitric oxide, can be prevented or modified by the action of inhibitors or antioxidants [27]. This is an area for future research trying to modulate this mechanism.

In fact, our study demonstrated that chronic stress also affects the antioxidant system, due to the low levels of GSH found in erythrocytes. Glutathione is a tripeptide composed of non-essential amino acids; these enzymes disintegrate the cellular protection system, favoring the establishment of an oxidative stress that can reach the periodontal defense metabolism [28]. Moreover, the lipid peroxidation (LPO) shown in CS and CS+EP groups can elucidate an aggravation in the microbial-initiated inflammatory process and it could be an important serum marker for both conditions [29].

In a molecular way, oxidative stress, promoted by the establishment of a chronic stress condition, modifies signaling events of different cells, including the regulation of mitogen-activated protein kinases (MAP kinases), intra-cellular calcium levels, and transcription factors [8,30,31]. In this sense, the production of EROS modulates bone metabolism from undifferentiated osteoclastic precursor cells, altering the activities of osteoblastic formation and osteoclastic bone resorption, favoring the creation of a pro-resorptive environment. The bone metabolism of osteoclastic and osteoblastic signaling was altered due to chronic stress, confirmed by the expression of the kappa B nuclear factor activator receptor (RANKL), which binds to the RANK receptor [32]. In addition to this factor, the literature points out that oxidative stress present in bone metabolism can affect the hematopoietic system and its contribution to innate immunity, acting on antigens as well as reaching differentiation of macrophages in osteoclasts [33].

Structurally, the remaining alveolar bone has also been modified due to changes in the numbers of trabeculae, as well as in the ratio between tissue volume and bone volume. The bone structure is dynamic, therefore, since bone formation activities (dependent on osteoblasts) and bone resorption events (promoted by osteoclasts) are unbalanced, with the establishment of a pathological condition in the alveolar bone [34]. Further, increased trabecular thickness, spacing and number per unit leading to a decreased percentage of bone volume. This research shows that CS promoted a pro-resorptive environment in periodontal tissues through an oxidative imbalance, which modulated the alveolar bone in a biological, physiological, and structural way.

In this sense, we highlighted the trabecular bone dynamism as an answer for this structural change. The trabecular tissue is essentially characterized by the presence of bone marrow (islets of hematopoietic tissue). In this sense, the bone marrow is qualified as a primary lymphoid tissue, which is responsible for the production of erythrocytes, granulocytes, monocytes, lymphocytes, and platelets, being an important source of stem cells for the alveolar bone. Thus, the presence of a larger trabecular network in the CS, EP, and CS + EP groups reflects the attempt of repair bone metabolism in the face of the damage caused by chronic stress and/or experimental periodontitis [35].

Morphologically, the alveolar bone loss induced by the presence of ligatures, which allows a microbiological challenge in EP and CS+EP groups, can potentially be explained by the fact that periodontal tissues tend to migrate to a more apical position, accompanying the bone resorption to recover the natural biological distances, in an attempt to decrease the severity of the disease; this has been demonstrated both in stereomicroscopic and microtomographic analyses, as it has been already reported in other studies in the literature [36,37]. Bone resorption is considered to be a way of trying to isolate the microbial challenge trying to prevent dissemination of the infectious material.

Currently, the impact of chronic stress on oral health, especially in clinical periodontics, is well reported, although the pathways involved in this process are rarely discussed in studies with humans [18,38,39,40,41]. It has been demonstrated that periodontal breakdown in rats has several similarities with humans; however, it is not identical. Genetic variability, specific anatomical differences of the oral cavity, and dependence of specific bacteria have been pointed out in experimental studies [42]. On the other hand, mechanistic studies are better performed in animals and should be part of the process of understanding. The present work has also demonstrated aspects that should be highlighted: masking of all assessors for group allocation, reproducibility of the measurements, and availability of identical conditions to the animals, except for variables of exposure. This study develops the generation of solid evidence to be fomented.

Another point to be raised in future studies on this theme refers to intervention models that limit the damage caused by chronic stress, as well as other adjuvant therapies that can modulate alveolar bone loss. Furthermore, future research investigating this condition should examine, for example, antioxidants therapy for modulation of this damage, such as vitamin C, green tea, or lycopene [13,43]. In this way, the scientific literature will allow a curative perspective of the morbidities that relate to periodontitis.

## 4. Methods

### 4.1. Animals and Experimental Design

The present study was a randomized controlled animal model experimental study. The Institutional Review Board of the Federal University of Pará (CEUA-UFPA) approved the protocol (4854071217, on 28 June 2018). Twenty-eight male Wistar rats (*Rattus norvegicus),* weighing 150–200 g were selected. The number of animals required for this experiment was calculated using the G * Power: Statistical Power Analyzes 3.1.9.2 program, with criteria for analysis of variances, based on the study in 2012 by Porto et al.; the effect size was 0.722, α err prob 0.05, and a power of 0.95 [44].

Animals were randomly allocated in four groups: control, EP, CS, and CS+EP (association between the experimental protocols). They were housed in polyethylene cages, with controlled food and filtered water, and maintained on a 12:12-h light/dark cycle.

**Figure 6:**
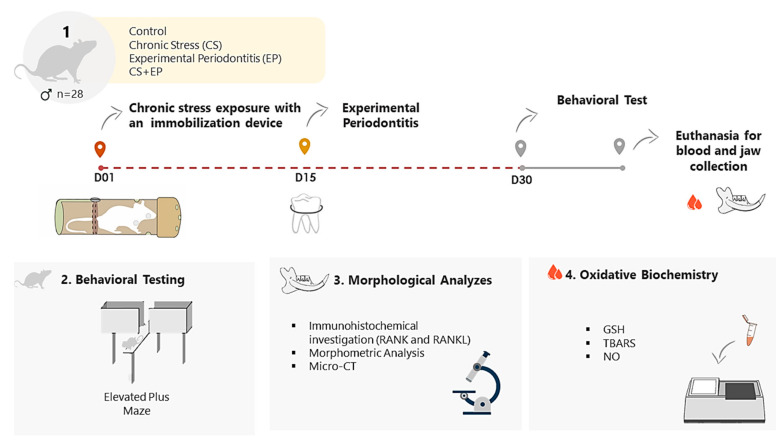
Methodological flow: (1) sample description and experimental steps; (2) elevated plus maze is the behavioral test adopted to investigate indirect parameters of chronic stress; (3) morphometric, microtomographic and immunohistochemical morphological analyzes in the hemi-mandibular bone. (4) Investigation of oxidative biochemistry determination of glutathione levels (GSH), lipid peroxidation (TBARS), nitric oxide (NO) levels.

CS exposure utilized a restrictive physical stress model [41] for 30 days in groups CS and CS+EP. In the same moment, the control and EP groups were maintained in the environment without CS exposure. On the 15th experimental day, the EP and CS+EP were submitted to ligature-induced periodontal breakdown. Ligatures were placed around lower first molars and were maintained until the end of the study. The control group received no intervention. The experimental design is described in Figure 6.

### 4.2. Chronic Stress Exposure

CS protocol was performed from the 1st day of the experiment on the CS and CS+EP groups up to the end of the experiment [18]. Exposure to chronic stress was performed by the method of immobilization/physical restraint. Therefore, animals were placed in polyvinyl tubes compatible with their size. These tubes were closed with a polyvinyl cap that allowed the adaptation of the animal length inside the tube, with holes to allow breathing for the animals inside the device. Animals were immobilized for a period of 4 h daily [41].

### 4.3. Experimental Periodontitis: Alveolar Bone Loss Induction

The EP induction was performed in the fifteenth day of the experiment. Ligatures remained until euthanasia [37]. For placement of the ligature in the EP and CS+EP groups, the animals were anesthetized by intraperitoneal injection with 2% xylazine hydrochloride (2 mg/mL) solution (Rompun^®^, Bayer Inc., Mississauga, ON, Canada) and 10% ketamine hydrochloride (10 mg/mL) (Dopalen^®^, Sespo Ltda., Paulínia, SP, Brazil) in the respective doses of 8 mg/kg and 75 mg/kg. Cotton ligatures were inserted on the cervical region of lower first molars of each animal. 

### 4.4. Behavioral Testing by Elevated Plus Maze (EPM) Analysis

The EPM was conducted between 10:00 AM and 3:00 PM in a sound-attenuated room under low-intensity light (12 lx), where the rats had been habituated for 2 h previous to the tests. The maze equipment was made of wood and consisted of four crossed arms, two closed (50 × 10 × 40 cm) and two open arms (50 × 10 × 1 cm), opposite each other and placed 50 cm above the floor. Each animal explored the equipment for 5 min to count the number of entrances in the closed arms and the percentage of open arm entries in relation to the total number of entries, as previously described [45].

### 4.5. Oxidative Biochemical Analysis

#### 4.5.1. Sample Preparation

Blood samples were collected in tubes containing 50 µL of 5% ethylenediamine tetra acetic acid (EDTA) and centrifuged at 3000 rpm for 10 min. After centrifugation, plasma and erythrocytes were collected and stored separately in microtubes. Plasma was stored at −80 °C and washed red cell suspensions with 0.9% saline with consecutive centrifugation 2500 rpm for 10 min (the procedure was repeated twice) to obtain 50% red blood cells, ready for frozen storage at −80 °C, for subsequent determination of enzyme analysis. The erythrocytes of the samples were analyzed for thiobarbituric acid reactive substances (TBARS), nitric oxide (NO), and reduced glutathione content levels (GSH) [46].

#### 4.5.2. Reduced Glutathione Content Measurements (GSH)

GSH level measurements were determined using a modified Ellman method [47]. First, the red blood cells were hemolyzed in cold distilled water. An aliquot (20 µL) of hemolysate was added to a tube containing distilled water (20 µL) and PBS-EDTA pH 8.0 buffer solution (3 mL) to perform the first measurement. Then 5,5-dithiobis (2-nitrobenzoic acid) (DTNB; 0.47 mmol) was added to the solution, and another measurement was performed after 3 min. GSH concentration was expressed in µg/mL.

#### 4.5.3. Measurement of Thiobarbituric Acid Reactive Substances (TBARS)

The determination of thiobarbituric acid reactive substances (TBARS) is an indicator of lipid peroxidation as described by Kohn and Liversedge [48] and modified by Percário [49]. Malondialdehyde (MDA) produced after the lipid peroxidation process reacts with thiobarbituric acid (TBA) and generates chromophore substance. Briefly, 1 mL of 10 nM TBA was added to 100 µL of samples after incubation for 1 h at 94 °C. Samples were cooled, *n*-butanol (4 mL) was added to each sample, and samples were homogenized and centrifuged at 2500 rpm for 10 min. The organic phase (3 mL) was read spectrophotometrically at 535 nm. TBARS concentration was expressed in µmol/L.

#### 4.5.4. Nitric Oxide (NO) Concentrations

NO was quantified as nitrate concentration by the Griess method [22]. Nitrate concentration in plasma samples was converted to nitrite by nitrate reductase. Briefly, 100 µL of samples were incubated with Griess reagent (100 µL) for 10 min at 37 °C. Absorbance was measured on a microplate reader (Spectra Max 250^®^, Molecular Devices Co., Menlo Park, CA, USA) at 550 nm. Nitric oxide concentration was determined in µmol [50].

### 4.6. Analysis of the Alveolar Bone Loss (ABL)

The mandibles were removed, divided into two sections, and fixed in 10% buffered neutral formalin solution for 48 h. The reduction of the pieces was performed carefully in order to keep the mandibular molars intact. The right hemimandibles were used to the evaluation of alveolar bone loss by stereomicroscopic and microtomographic analyses while those on the left side were directed for immunohistochemical analysis.

The right hemi-jaws were dissected, and the anatomical parts obtained were individually packed in plastic pots containing 10% buffered neutral formalin solution. The left hemi-jaw samples were decalcified in EDTA solution. They were kept immersed in the solution, and the substance was renewed daily during the descaling period. Decalcification was controlled by attempting to transfix the specimen with a histological needle.

#### 4.6.1. Immunohistochemical Investigation for RANK and RANK-L

To analyze bone metabolism through the investigation NF-κB (RANK) and receptor activator of the NF-κB ligand (RANKL), the hemimandible tissue was deparaffinized and rehydrated. Gingival and periodontal tissue slices (4 µm) were washed with 0.3% Triton X-100 in phosphate buffer, quenched with endogenous peroxidase (3% hydrogen peroxide), and incubated overnight at 4 °C with primary antibodies against the following proteins (all antibodies 1:400): RANK and RANKL (INTERPRISE^®^, Santa Cruz Biotechnology, São Paulo, SP, Brazil) and incubated for 30 min with a streptavidin/horseradish peroxidase conjugated to secondary antibody (Biocare Medical^®^, Concord, CA, USA). Immunostaining was visualized using colorimetric detection (Biocare Medical^®^, Dakota, ND, USA). The staining status was identified as either negative or positive; positive staining was defined as the presence of brown chromogen. Staining intensity and the proportion of immunopositive cells were examined independently by two pathologists using light microscopy and recorded. Intensity of staining (IS) was graded on a 0 to 2 scale according to the following semi-quantitative assessment: 0 = no detectable staining, 1 = weak staining; 2 = strong staining [51].

#### 4.6.2. Micro-CT Analysis

Animals’ hemimandible were submitted to micro-CT (MicroCT.SMX-90 CT; Shimadzu Corp., Kyoto, Japan). Each hemi-mandible was placed on a rotatory platform inside the equipment. Images were captured under a rotation of 360°, with an intensity of 70 kV and 100 mA. After this, images were reconstituted by inspeXio SMX-90CT software (Shimadzu Corp., Kyoto, Japan), with a 10 µm voxel size in images with resolution of 1024 × 1024 and thickness of 14 µm, which resulted in 541 images per sample. Bone loss in height evaluation was performed on RadiAnt DICOM Viewer 5.0.1 (Medixant, Poznan, Poland) software for the 3D reconstruction of the hemimandible. The tridimensional models were placed on a standard position, where the lingual tooth face could be observed. Thus, the vertical bone loss was detected through the measurement of the distance between the cementum–enamel junction and the alveolar bone crest at three points of the first inferior molar (i.e., mesiolingual, midlingual, distolingual), performing the average of these regions. 

In order to verify the alveolar bone tissue quality, ImageJ^®^ (National Institutes of Health, Bethesda, MD, USA) software was used in a selected stack of 70 images from the inferior first molar alveolar bone region. The interradicular region, close to the furcation area, from the inferior first molar, including the cervical third until the middle third of the root (average area of 0.200 mm^2^), was chosen as the standardized region of interest (ROI).

A threshold was applied to the segmentation of the different scores of gray color present in the image. To select the threshold, the differences between the bone gray values and the gray values of other structures were considered. Based on these, the threshold was set in at 150 to 255. Employing the plug-in BoneJ, the trabecular thickness (Tb.Th), trabecular spacing (Tb.Sp), trabecular number (Tb.N), and bone fraction (%BV/TV) were evaluated. The measurements were performed by one calibrated examiner that was submitted to an interclass correlation coefficient (ICC) test prior to the analysis [52,53].

#### 4.6.3. Morphological Analyses

The hemimandibles of each group were observed in a stereomicroscope (Discovery V8 Zeiss, Jena, Germany). The samples were immersed with 8% sodium hypochlorite (NaOCl) for 4 h, after washing with distilled water in an ultrasonic bath for 10 min. 

To differentiate the cementum enamel junction (CEJ), the samples were immersed in 1% methylene blue for 60 s, followed by washing with water. The specimens were then dried at room temperature and prepared (wax-fixed), whose jaw lingual surface was perpendicular to the axis of observation, and examined using a reticulum chart attached to the stereomicroscope of the eyepiece.

Images were taken with a 6.1-megapixel camera (Canon Powershot A640; Canon, Lake Success, NY, USA) coupled to the stereomicroscope (3.2×). Using stereomicroscopic images, ABL was measured by the distance between the CEJ and the alveolar bone crest of the first molars lingual face, using the unit area (mm²) [37].

### 4.7. Statistical Analysis

In this research, the results were tabulated and analyzed by GraphPad Prism 7.0 software (GraphPad Software Inc., La Jolla, CA, USA). The Shapiro–Wilk test was adopted to verify the normality; the data was normal if the *p*-value was above 0.05. Variables with a non-normal distribution were analyzed by the Kruskal–Wallis test. In variables displaying a normal distribution, the one-way ANOVA test was performed with the Tukey post-test, considering a significant value of *p* < 0.05.

## 5. Conclusions

Based on the results of this study, chronic stress induces oxidative blood imbalance through an increase in the levels of nitric oxide and a decrease in the concentration of GSH, potentiating the morphological and metabolic damage to the alveolar bone, both in the presence and in the absence of experimental periodontitis. Chronic stress generates an anxiety pattern that exacerbates periodontal breakdown.

## Supplementary Materials

The supplementary table with all data can be found at https://www.mdpi.com/1422-0067/21/10/3728/s1. Table S1: Quantification data for all analysis carried out.

## Abbreviations

ABC	Alveolar bone crest
CEJ	Cementoenamel junction
CS	Chronic stress
EDTA	Ethylenediamine tetraacetic acid
EPM	Elevated plus maze
EP	Experimental periodontitis
GSH	Glutathione
IHC score	Immunohistochemistry score
NO	Nitric oxide
RANK	NF-κB
RANKL	NF-κB ligand
TBARS	Thiobarbituric acid reactive substances levels
