# Supplementary material for: Blood Oxidative Stress Modulates Alveolar Bone Loss in Chronically Stressed Rats
Source: Int J Mol Sci. 2020 May 25;21(10):3728. doi: 10.3390/ijms21103728 (PMC7279478; doi:10.3390/ijms21103728)
Supplement: Supplementary file 1 [file ijms-21-03728-s001.pdf]

International Journal of *Molecular Sciences*

MDPI

Supplementary Material

Blood Oxidative Stress Modulates Alveolar Bone Loss in Chronically Stressed Rats

Micaele Maria Lopes Castro [1], Priscila Cunha Nascimento [1], Deiweson Souza-Monteiro [1], Sávio Monteiro Santos [2], Mayra Barros Arouck [3], Vinicius Barreto Garcia [4,5], Raimundo Fernandes de Araújo, Jr. [4,5,6,7], Aurigena Antunes de Araujo [2], Gabriela de Souza Balbinot [8], Fabrício Mezzomo Collares [8], Cassiano Kuchenbecker Rosing [9], Marta Chagas Monteiro [2], Cristiane Socorro Ferraz Maia [3] and Rafael Rodrigues Lima [1,*]

[1] Laboratory of Functional and Structural Biology, Institute of Biological Sciences, Federal University of Para, 66075-900 Belem, PA, Brazil; micaelecastro@hotmail.com (M.M.L.C.); priscilacunha.n28@gmail.com (P.C.N.); deiwesonmonteiro@gmail.com (D.S.-M.)

[2] Laboratory of in vitro Assays, Immunology and Microbiology, Faculty of Pharmacy, Institute of Biological Sciences, Federal University of Pará, 66075-900 Belém, PA, Brazil; saviomontsan@gmail.com (S.M.S.); martachagas2@yahoo.com.br (M.C.M.); auriprinino@gmail.com (A.A.d.A.)

[3] Laboratory of Pharmacology of Inflammation and Behavior, Faculty of Pharmacy, Institute of Health Science, Federal University of Pará, 66075-900 Belém, PA, Brazil; mayraarouckbarros@gmail.com (M.B.A.); crismaia@ufpa.br (C.d.S.F.M.)

[4] Cancer and Inflammation Research Laboratory, Department of Morphology, Federal University of Rio Grande do Norte 59078-970 Natal, RN, Brazil; araujojr@cb.ufrn.br (V.B.G.); araujojr.morfologia@gmail.com (R.F.d.A.,Jr.)

[5] Post-Graduation Programme in Health Science, Federal University of Rio Grande do Norte, Natal, RN 59072-970, Brazil

[6] Post-Graduation Programme in Structural and Functional Biology, Federal University of Rio Grande do Norte, Natal, RN 59072-970, Brazil; (araujojr.morfologia@gmail.com)

[7] Department Biophysics and Pharmacology, Post-Graduation Programme in Public Health, Post-Graduation Programme in Pharmaceutical Science, Federal University of Rio Grande do Norte, Natal, RN 59072-970, Brazil

[8] Dental Materials Laboratory, Faculty of Dentistry, Federal University of Rio Grande do Sul, 90040-060 Porto Alegre, Brazil; gabi_balbinot@hotmail.com (G.d.S.B.); fabricio.collares@ufrgs.br (F.M.C.)

[9] Department of Periodontology, Faculty of Dentistry, Federal University of Rio Grande do Sul, 90040-060 Porto Alegre, Brazil; ckrosing@hotmail.com (C.K.R.)

* Correspondence: rafalima@ufpa.br or rafaelrodrigueslima@hotmail.com; Tel.: +55 91 3201 7891

This Supplementary Material contains 1 table.

International Journal of *Molecular Sciences*

Table S1. Quantification data for all analysis carried out presented in mean (±standard error), n= 7 per group.

Measures	Control Group	Experimental Periodontitis	Chronic Stress	Experimental Periodontitis + Chronic Stress	*p*-value
Rearings (n)	2.5 ± 0.37[a]	3 ± 0.51[a]	6.35 ± 0.75[b]	7.5 ± 1.44[b]	<0.05
Total time of grooming (s)	36.25 ± 2.5[a]	44.8 ± 1.2[a]	63.8 ± 5.46[b]	69.25 ± 10.45[b]	
Open arms entries (%)	47.5 ± 5.83[a]	36.55 ±6.48[a]	8.33 ± 8.33[b]	4.16 ± 4.16[b]	
Time in open arms (%)	6.66 ± 1.87[a]	5.6 ± 1.37[a]	1.2 ± 0.79[b]	0.96 ± 0.56[b]	
Alveolar bone-loss area (mm2)	1.72 ± 0.01[a]	2.53 ± 0.1[b]	2.24 ± 0.1[b]	3.12 ± 0.09[c]	
Distance between the CEJ and ABC (mm)	0.86 ± 0.01[a]	1.08 ± 0.02[b]	1.04 ± 0.04[b]	1.22 ± 0.03[c]	
Tb.Th (mm)	0.1 ± 0.01[a]	0.11 ± 0.01[ab]	0.14 ± 0.01[b]	0.19 ± 0.01[c]	
Tb.Sp (mm)	0.13 ± 0.01[a]	0.21 ± 0.01[bc]	0.18 ± 0.02[ac]	0.3 ± 0.02[d]	
Tb.N (mm)	0.23 ± 0.01[a]	0.33 ± 0.01[b]	0.32 ± 0.02[b]	0.49 ± 0.02[c]	
BV/TV (%)	0.39 ± 0.01[a]	0.26 ± 0.02[b]	0.26 ± 0.03[b]	0.2 ± 0.01[b]	
IHC score RANK	0 (0-0.25)[a]*	2 (1.75-2.25)[b]*	2 (2-3)[b]*	2 (1.75-2)[b]*	
IHC score RANKL	0 (0-0)[a]*	0 (0-0.25)[a]*	1 (0-1)[a]*	1 (1-1.25)[a]*	
GSH levels (% of control)	100 ± 1.95[a]	79.6 ± 1.62[b]	70.13 ± 2.38[b]	58.4 ± 5.37[c]	
NO concentration (% of control)	100 ± 7.53[a]	243 ± 22.5[b]	245.7 ± 25.54[b]	335 ± 19.04[c]	
TBARS levels (% of control)	100 ± 3.03[a]	132.7 ± 3.14[b]	161.5 ± 6.13[c]	180.4 ± 2.85[d]	

Similar overwritten letters did not show significant statistical differences (One-way ANOVA and Tukey’s post-hoc test); CEJ: cementoenamel junction; ABC: alveolar bone crest; IHC score: immunohistochemistry score; GSH: glutathione; NO: nitric oxide; TBARS: thiobarbituric acid reactive substance levels; *results expressed as median and interquartile range (Kruskal-Wallis test).
